# Social differences in diagnosed depression among adolescents in a Swedish population based cohort

**DOI:** 10.1186/s12888-018-1765-0

**Published:** 2018-07-03

**Authors:** Therese Wirback, Jette Möller, Jan-Olov Larsson, Karin Engström

**Affiliations:** 1 Department of Public Health Sciences, Karolinska Institutet, Solnavägen 1E, 113 65 Stockholm, Sweden; 2 Department of Women’s and Children’s Health, Karolinska Institutet, Tomtebodavägen 18A, 171 77 Stockholm, Sweden

**Keywords:** Socioeconomic status, Cohort study, Depression, Adolescence, Gender

## Abstract

**Background:**

Population based research regarding social differences in diagnosed depression in adolescence is sparse. In this study unique material containing in-and outpatient data was used to determine if low social position in childhood increases the risk of diagnosed depression in adolescence. To further examine this association, gender differences and interactions were explored.

**Methods:**

The study population was extracted from the Stockholm Youth Cohort (SYC), a register based cohort containing psychiatric care for all young people in Stockholm County and information about social position. For the purpose of this study, all in the SYC who turned 13 years old during 2001–2007, in total 169,262 adolescents, were followed up in 2005–2011 for diagnoses of depression until age 18. Associations were estimated with Cox regression models and presented as Hazard Ratios (HR).

**Results:**

The risk of diagnosed depression was higher for adolescents with parents with low education (HR = 1.1, CI = 1.0–1.2) and medium education (HR = 1.1, CI = 1.1–1.2) compared to high as well as for those with lower household income (for example, medium low, HR = 1.2, CI = 1.1–1.3) and for those with parents who received an unemployment benefit (HR = 1.3, CI = 1.2–1.4). No differences were found for those with the lowest household income compared to those with the highest level. Adolescents with parents born outside the Nordic countries had a lower risk of diagnosed depression (HR = 0.7, CI = 0.6–0.7). An interaction effect was found between gender and parental education.

**Conclusions:**

Social differences were found but the magnitude was modest and gender differences small.

## Background

Depression is a leading cause of years lived with disabilities (YLDs) [1, 2] and often originates early in life [3, 4]. The prevalence of adolescents treated for depression in Sweden has increased during the past two decades, and is more common among females [5]. Much research points at higher risks of poor mental health among adolescents with low social position [e.g. 6–18], though some studies found no association [19, 20]. There are likely several mechanisms behind social differences, including differential exposure to determinants of depression [21, 22] as well as negative psychosocial factors which can cluster in persons with low social position. How people describe depressive symptoms and where people seek care can further vary depending on for example academic background [23, 24]. Moreover, children of parents with depression can be at a higher risk for depression [25] both because of social and genetic influence. Overall, the results of studies investigating social differences in depression are hard to summarize due to methodological differences such as the measures used to assess depression as well as social position [7, 9, 11, 14, 15, 17–20, 26, 27]. So far most studies on social position and poor mental health in adolescence have been based on psychiatric disorders overall [14, 16, 28, 29], poor mental health overall [6, 8, 10, 12, 13, 16, 30, 31], or self-reported depressive symptoms [7, 9, 11, 15–17, 19, 26, 32]. Only a few studies focus on diagnosed depression or mood disorders specifically [18, 20, 27, 33]. McLaughlin et al. [18] show in their study from the United States that financial hardship in childhood increases the risk of diagnosed mood disorders in adolescence, but low parental education and lower status occupation do not. A Finish study with a focus on late adolescence reports that both low parental income and education increase the risk of depression, but education to a lesser extent [27]. Two less recent studies, one from New Zealand and one from the United States, found no increased risk of depression [20] or mood disorders [33] among groups with low socioeconomic status. Gender-specific risks in relation to social position and mental health problems show inconclusive results [16]. Some studies indicate that girls are more vulnerable [17, 30, 34] whereas others point to boys’ vulnerabilities [10, 31, 32], and some report no gender differences [6, 11, 15]. Only one study looked at gender differences in relation to social position and diagnosed depression [27], and they found merely small differences.

To add to the limited knowledge of social differences in diagnosed depression among adolescents - in a setting where free mental health services are available - this Swedish population based cohort study was performed. It will also increase knowledge on whether boys and girls are equally affected. Registers with several measures of social position and with diagnoses set by professionals within clinical settings was used. The aim was to determine if low social position in childhood increases the risk of diagnosed depression in adolescence (13–17 year olds), and if there are gender-specific differences.

## Methods

### Setting and material

The material derives from Sweden, a country with a relatively strong redistributive system with mental health services for adolescents primarily financed by tax. The public Child and Adolescent Mental Health Service (CAMHS; In Swedish, BUP) is free of charge for the patient. Children and adolescents can be referred to CAMHS from primary care, seek care directly themselves or via their parents. Primary care with public general practitioners, counsellors and therapists, internet-based psychiatric help and psychiatric emergency units (free for children and adolescents) is also available, as well as private therapists (high fee but mainly targeting adults).

This study is based on The Stockholm Youth Cohort (SYC), a register-based cohort containing information of psychiatric care of children and adolescents in Stockholm County Council (SLL) as well as several measures of social position. SYC includes all individuals aged 0–17 residing in Stockholm County at any point in time during 2001 and 2011, ascertained from the Swedish register of the total population [35]. The SYC is built up with linkage to several different health care and administrative registers by the personal identification number for each individual (recoded for research use). The SYC database is held by the Department of Public Health Sciences at Karolinska Institute. The registers used for the current study were: CAMHS, which includes public psychiatric in-and outpatient care in Stockholm County; The Cause of Death Register [36] with dates and causes of death, according to ICD (International Classification of Disease), for Swedish residents; The National Patient Register which initially included all patients treated in psychiatric care and some somatic care at public hospitals, but now includes information about all public in-patient care (from 1984) and out-patient visits to specialized clinics at hospital (from 2001) in Sweden [37], but not primary care; health care registers in SLL (VAL, Swedish acronym) that include in- and outpatient care for residents in Stockholm County, including public and private outpatient care clinics and primary care facilities as well as hospitals for public and private inpatient care, financed by SLL but not private specialists [38]; The Longitudinal Integration Database for Health Insurance and Labour Market Studies (LISA, Swedish acronym) [35], with information about employment, education, income and social benefits for those over 16 years old residing in Sweden; The Multi-generation Register which is part of the Total Population Register and includes connections between index persons and their parents [39].

### Study population and design

The study population was extracted from SYC and is defined as adolescents who were residing in Stockholm County the year they turned 13, between 2001 and 2007, i.e. 7 consecutive birth cohorts (born 1988–1994). A total of 169,262 adolescents were followed for 5 years, until they turned 18, in 2005–2011. A closed cohort design was employed. Individuals were censored at whatever occurred first: time of first depression diagnosis, death, moving out of Stockholm County or end of follow up at age of 18. Age was restricted to 18 as individuals in the Swedish health care system are then considered adults and shall seek care at mental health services for adults.

### Exposure and outcome

#### Social position

Parental education and household disposable income was used to assess childhood social position. To capture additional aspects of social position, parental receipt of an unemployment benefit and parental country of origin were assessed. Parents were defined as social parents, i.e. persons that the child lives with, including biological parents, adoptive parents and other adults responsible for the child. *Parental education* was assessed according to the Swedish standard for classification of individual educational programs [40] and was categorized in three groups; low (pre-secondary), medium (upper secondary), and high (post-secondary) based on the parent with the highest education. *Household disposable income* [35] is an individualized weighted average income, i.e. the sum of the family members’ disposable income multiplied by the individual consumption weights and divided by their aggregate consumption weight. It was categorized according to the distribution within the study population in quintiles; low, medium-low, medium, medium-high and high. *Receipt of an unemployment benefit* was defined and categorised as, two, or the single parent receiving it once or several times, or not. *Parental country of birth* was categorised into born in Sweden, born in the Nordic countries (but not in Sweden), or born outside the Nordic countries. Adolescents were classified as having non-Swedish parents if the lone or both parents were born outside of Sweden, where the parental country of birth geographically closest to Sweden was used in the categorization. Born in Sweden was the reference category. All measures of social position were assessed at age 12, the year before the start of follow up, and collected from LISA.

#### Depression

Depression was defined as a first time depression diagnoses registered at CAMHS or in VAL. Diagnoses are clinical and were not established specifically for the aim of this study. Depression was defined as to include major depressive disorder, unspecified depressive disorder and dysthymic disorder (DSM-IV: 296.20–24, 300.4, 296.30–36, 311 including the DSM-IV based diagnostic code 19 used at CAMHS; ICD-10: F32.0–9, F38.0–8, F39, F33.0–9, F34.8–9).

### Confounding and mediation

Parental depression was considered a potential confounder since previous research have shown that children of parents with depression have a higher risk for depression [25], and a history of depression has been found to confound the relationship between SES (Socioeconomic Status) and major depression among adults [41]. Information regarding parental depression was collected from the National Patient Register and was taken into consideration if it occurred before the child turned 13. Parental depression was defined as occurring if a parent received a diagnosis in in-or outpatient care according to the following diagnoses from ICD 8, 9 and 10; F32.0–9, F33.0–9, F34.1,8,9, F38.0,1,8, F39, 300E, 296B, 311, 300, 296, 301.10, 302.99, 314.99. In the current study 4% had a mother or father with depression. Measures of social position and psychosocial factors, e.g. childhood adversities such as divorce, which are considered to occur within the causal pathway, were not adjusted for.

### Statistical analysis

The Incidence Rate Ratio (IRR) and corresponding 95% confidence intervals (CI) of social position and first time onset of diagnosed depression were calculated using Cox regression models and are presented as Hazard Ratios (HR). Boys and girls were analysed jointly as well as separately to explore possible gender differences. Estimates were calculated for all measures of social position separately. Additionally, adjustments were included for parental depression, country of birth, and education. A test for fulfilment of the proportional hazards assumption was performed with a time dependent variable [42]. There was no indication of a violation of the assumption. Additive interactions were calculated by using the Synergy Index (SI) [43, 44]. SI calculations require dichotomized independent variables. For this purpose the following variables were dichotomized: parental education (low/medium vs. high), household income (low/medium low/medium vs. medium high/high) and parental country of birth (born in Sweden or not). To make use of as much information as possible in the respective analyses, individuals with missing information on respective exposure variable were excluded only for that particular analysis. A sensitivity analysis including complete cases showed no significant differences. All analyses were done using SAS version 9.3 and 9.4 (SAS Institute Inc. Cary, N.C., US.

## Results

Most adolescents had parents with high education level and 9% had parents with a low level, which corresponds quite well with the national average [45]. Six percent had received an unemployment benefit and 19% had parents born outside the Nordic countries while 3% had parents born in Nordic countries. Characteristics of the adolescents are presented in Table 1.

**Table 1:** Characteristics among the adolescents, with and without diagnosed depression, and by gender

	All (*n* = 169,262)		Boys (*n* = 86,888) 51.3%		Girls (*n* = 82,374) 48.7%	
	Depression (*n* = 6439)	No depression (*n* = 162,823)	Depression (*n* = 1978)	No depression (*n* = 84,910)	Depression (*n* = 4461)	No depression (*n* = 77,913)
	%		%		%	
Parental country of origin						
Sweden	67.2	63.4	66.6	63.2	67.4	63.6
Nordic	3.3	2.9	2.8	3.0	3.5	2.8
Outside Nordic	13.2	19.7	14.7	19.7	12.6	19.7
Missing	16.3	14.1	15.9	14.2	16.5	14.0
Parental education						
High	44.9	47.7	46.3	47.8	44.3	47.6
Medium	44.2	41.0	43.4	41.0	44.5	41.0
Low	9.2	9.4	8.3	9.3	9.6	9.4
Missing	1.7	2.0	2.0	2.0	1.6	2.0
Disposable household income						
High	16.2	18.2	15.1	18.2	16.7	18.3
Medium high	20.2	19.7	21.2	19.7	19.7	19.7
Medium	22.5	20.0	23.8	20.0	21.9	20.0
Medium low	22.4	20.4	21.8	20.5	22.7	20.4
Low	17.5	20.4	16.7	20.4	17.8	20.5
Missing	1.3	1.3	1.3	1.3	1.2	1.3
Parental receipt of an unemployment benefit						
Not received	86.3	86.1	85.1	86.1	86.8	86.0
Received	6.7	5.3	7.5	5.4	6.4	5.3
Missing	7.0	8.6	7.4	8.6	6.8	8.7

During follow-up, 6439 adolescents, 1978 boys (2.3%) and 4461 girls (5.4%), were diagnosed with first time depression. The majority of the cases of depression were identified in the outpatient clinics. Of the adolescents with depression, 242 (3.7%) had been treated in in-patient care (0.14% of the total study population); 84 boys (4.2% of the depressed boys) and 158 girls (3.5% of the depressed girls). The mean age for diagnosis of first time depression was 15.36 for girls and 15.38 for boys. Depression was more common in later adolescence, especially for girls. The largest proportion received a diagnosis at the age of 17 (27%) and the smallest at age 13 (12%). Most adolescents with depression were diagnosed with the CHAMS code 19 (69.4%), followed by major depressive disorder (23.4%), unspecified depressive disorder (6%) and dysthymic disorder (1.2%).

### Childhood social position and risk of diagnosed depression in adolescence

Crude and adjusted HRs are shown in Tables 2 and 3. The risk of depression diagnosis was slightly higher for adolescents with parents of a low and medium education compared to high (HR = 1.1). Increased risks were found for adolescents with medium-low (HR = 1.2), medium (HR = 1.3), and medium-high household income (HR = 1.2) when compared to those with the highest. No increased risk was found for those with the lowest income compared to high. Having parents who received an unemployment benefit was associated with a higher risk of diagnosed depression (HR = 1.3). Adolescents with parents born outside the Nordic countries had a lower risk of diagnosed depression (HR = 0.7), whereas those with parents born in the Nordic countries had a marginally increased risk (HR = 1.1) compared to those with Swedish-born parents. The effect estimates remained or decreased marginally when adjusted for parental depression. Results with adjustments for parental depression are therefore only shown in the first model. Overall, the effect estimates increased slightly when adjusting for parental country of origin and education.

**Table 2:** Crude and adjusted Hazard Ratios (HR) of diagnosed depression in adolescence, by social position, *n* = 169,262

Social factors	Crude HR (95% CI)	Adjusted for biological parents depression HR (95% CI)	Adjusted for parental country of origin HR (95% CI)	Adjusted for parental country of origin and education HR (95% CI)
Sex				
Boy	1.0			
Girl	2.4 (2.3–2.6)			
Parental country of origin				
Sweden (ref.)	1.0			
Nordic	1.1 (1.0–1.3)			
Outside Nordic	0.7 (0.6–0.7)			
Parental education				
High (ref.)	1.0	1.0	1.0	
Medium	1.1 (1.1–1.2)	1.1 (1.1–1.2)	1.2 (1.1–1.3)	
Low	1.1 (1.0–1.2)	1.0 (0.9–1.1)	1.2 (1.1–1.4)	
Disposable Household income				
High (ref.)	1.0	1.0	1.0	1.0
Medium high	1.2 (1.1–1.2)	1.1 (1.1–1.2)	1.2 (1.1–1.3)	1.2 (1.1–1.3)
Medium	1.3 (1.2–1.4)	1.3 (1.2–1.4)	1.3 (1.2–1.5)	1.3 (1.2–1.4)
Medium low	1.2 (1.1–1.3)	1.2 (1.1–1.3)	1.4 (1.2–1.5)	1.3 (1.2–1.4)
Low	1.0 (0.9–1.1)	1.0 (0.9–1.1)	1.2 (1.0–1.3)	1.1 (1.0–1.2)
Parental receipt of an unemployment benefit				
Not received (ref)	1.0	1.0	1.0	1.0
Received	1.3 (1.2–1.4)	1.2 (1.1–1.4)	1.4 (1.3–1.6)	1.4 (1.2–1.5)

**Table 3:** Gender stratified Hazard Ratios (HR) of diagnosed depression in adolescence, by social position, *n* = 169,262

Social factors	Crude HR (95% CI)		Adjusted for parents country of origin HR (95% CI)		Adjusted for parents country of origin and education HR (95% CI)	
	Boys	Girls	Boys	Girls	Boys	Girls
Parental country of origin						
Sweden (ref.)	1.0	1.0				
Nordic	0.9 (0.7–1.2)	1.2 (1.0–1.4)				
Outside Nordic	0.7 (0.6–0.8)	0.6 (0.6–0.7)				
Parental education						
High (ref.)	1.0	1.0	1.0	1.0		
Medium	1.1 (1.0–1.2)	1.2 (1.1–1.2)	1.2 (1.0–1.3)	1.2 (1.1–1.3)		
Low	0.9 (0.8–1.1)	1.1 (1.0–1.2)	1.1 (0.9–1.3)	1.3 (1.2–1.5)		
Disposable household income						
High (ref.)	1.0	1.0	1.0	1.0	1.0	1.0
Medium high	1.3 (1.1–1.5)	1.1 (1.0–1.2)	1.4 (1.2–1.6)	1.1 (1.0–1.3)	1.4 (1.2–1.6)	1.1 (1.0–1.2)
Medium	1.4 (1.2–1.7)	1.2 (1.1–1.3)	1.5 (1.3–1.8)	1.3 (1.1–1.4)	1.5 (1.3–1.8)	1.2 (1.1–1.3)
Medium low	1.3 (1.1–1.5)	1.2 (1.1–1.3)	1.5 (1.2–1.7)	1.3 (1.2–1.5)	1.4 (1.2–1.6)	1.3 (1.1–1.4)
Low	1.0 (0.9–1.2)	1.0 (0.9–1.1)	1.2 (1.0–1.4)	1.2 (1.0–1.3)	1.1 (0.9–1.3)	1.1 (1.0–1.2)
Parental receipt of an unemployment benefit						
Not received (ref)	1.0	1.0	1.0	1.0	1.0	1.0
Received	1.4 (1.2–1.7)	1.2 (1.1–1.4)	1.5 (1.3–1.8)	1.4 (1.2–1.6)	1.5 (1.2–1.8)	1.3 (1.2–1.5)

### Gender differences

Girls had a more than twofold increased risk of diagnosed depression compared to boys (HR = 2.4, CI = 2.3–2.6). Gender stratified associations between social position and diagnosed depression showed minor differences. Risk estimates were slightly higher for boys with a lower household income and parental receipt of an unemployment benefit, and for girls with higher parental education; however with overlapping CIs. See Table 3.

Additive interaction effects of gender and different measures of social position are shown in Table 4. Being a girl and having low educated parents had a synergistic effect of 1.2 (CI = 1.0–1.4).

**Table 4:** Hazard Ratios (HR) and Synergy Index (SI) between gender and social position in diagnosed depression, *n* = 169,262

Social factors	Male (95% CI)		Female (95% CI)		Synergy Index (SI) (95% CI)
	Unexposed to social factor	Exposed to social factor	Unexposed to social factor	Exposed to social factor	
Parental education^a^ High vs. low/medium	1.0	1.1 (1.0–1.2)	2.3 (2.0–2.6)	2.7 (2.5–3.0)	1.2 (1.0–1.4)
Disposable household income^b^ High vs. low	1.0	1.0 (0.9–1.1)	2.3 (2.2–2.5)	2.5 (2.3–2.7)	1.1 (0.9–1.3)
Parental receipt of an unemployment benefit^b^ Received vs. not received	1.0	1.4 (1.2–1.7)	2.3 (2.2–2.5)	3.1 (2.7–3.5)	1.2 (0.9–1.5)

^a^Adjusted for parental country of origin

^b^Adjusted for parental country of origin and education

## Discussion

The findings from this population based cohort study from Sweden expand upon and handle some methodological shortcomings of previous research. In the current study it was shown that 3.8% of 13–17 year old adolescents received a diagnosis of depression during a follow-up period of 5 years. The risk of diagnosed depression was higher for adolescents with parents who hold a medium education level, have a lower household income, received an unemployment benefit or were born in the Nordic countries. The social differences were however modest and not found for adolescents with the lowest household income. In addition, adolescents with parents born outside the Nordic countries had a lower risk of diagnosis. No prominent gender differences were found.

The increased risk of diagnosed depression for adolescents with a lower household income or with parents with a lower level of education, found in the current study, is in line with results from recent research from Finland and the US. In comparison however, the current study found smaller differences over all, and no increased risk for those with the very lowest income. The latter contrasts the Finnish study, and an increased risk for those in the group with the lowest income was also found in another Finnish study, where self-reported depressive symptoms were studied [46]. This indicates possible national differences, despite the fact that Finland has a welfare state similar to Sweden. Larger social differences, related to education, have also been noted in another study from Sweden, where self-reports of depressive symptoms were studied [17], which could indicate that groups with low social position seek care to a lower extent. The increased risk of depression for adolescents with parents who received an unemployment benefit are also in line with previous studies [17, 47, 48]. McLaughlin et al. [18] suggests that material deprivation such as financial hardship has a stronger association than relative status, as indicated by education for example, which is supported by the results from the current study as social differences related to low income and receipt of unemployment benefit were more prominent than social differences related to education.

One issue that can explain modest social differences in adolescence, as seen in this study, is the theory of social equalization in adolescence. The theory implies that adolescence is the life period when social differences are expected to be the smallest [13, 49]. Adolescence is a period when youths interact a lot with friends and thereby start to develop their own social position, whereas the social position of younger children depends more on their parents’ social position. Differences in the magnitude of social inequalities between different studies cannot be fully explained by this. The Swedish context, with relatively low social differences overall, in combination with a possible social equalization in adolescence, can however add to the explanation as to why a smaller magnitude of social differences were found in the current study. The widespread, and free access to mental health services can further add to the explanation. In addition, it has been shown that the influence of equalization in adolescence can depend on the specific health outcome as well as the measure used for social position [50, 51].

The protective effect of having parents born outside the Nordic countries contrasts earlier findings from a similar population in Sweden, focusing on self-reported depressive symptoms, where no differences between foreign-born and Swedish-born were detected [17, 52]. This indicates that adolescents with parents born outside the Nordic countries may seek care to a lower extent. A previous Swedish study reports that adolescents with parents born in in middle or low-income countries use psychiatric care to a lesser extent than native-born Swedes [53]. Possible explanations can include problems in accessing mental health services or lower health literacy; both in comparison with those whose parents are born in Sweden and in the Nordic countries since Nordic nations have more similar health systems and traditions. Differences can also be related to how depression is expressed and articulated in different cultures [54]. More work is needed to elucidate social differences in care-seeking for depressive symptoms, especially for foreign-born.

As expected, and in line with previous research, both on self-reported depressive symptoms [12, 17, 55] and diagnosed depression [27, 33], girls had an increased risk compared to boys. That girls suffer more from depression can partly depend on the diagnostic criteria or that expressions of depressive symptoms differ between boys and girls [56]. Boys can for example have succumbed to traditional norms of masculinity and not express their symptoms [57]. Regarding the gender-specific risks in relation to social position, the current study support to some extent the findings from studies that did not detect gender differences [6, 11, 15]. Muntaner [58] mention that different SES indicators can perform inconsistently for men and women, and even though only small gender differences were found in the current study there was some indication of inconsistency depending on the SES indicator. In the current study girls living with low educated parents were found to be somewhat more vulnerable for depression, which parallels the findings from one of the research group’s previous studies [17] on depressive symptoms. There are contradictory theoretical models to explain an elevated risk of depression depending on social position, for boys and girls respectively. It has been suggested that low parental education is a more powerful risk factor for depression among boys than girls [59]. Girls may not be affected as much as boys by the added vulnerability of low parental education because they are already so vulnerable to depression. However our results support the theoretical model proposed by Cyranowski et al. [60], which proposes that girls are more vulnerable to depression due to the combination of a more affiliative style in social relations and transition difficulties, which are triggered by negative life events. Further, social position can have different meanings and effects on mental health depending on for example the context and ethnicity [61, 62], which highlights the importance of studies from different countries.

A complex picture of the association between social position and depression is revealed, and it reminds us that the indicators used to define social differences describe different dimensions of social position [63]. Income translates into material and immaterial resources and fluctuates to some extent, whereas education is more stable and relates to, for example, health literacy. These different dimensions are considered to be interconnected, and the cause and effect chain depends on what is being studied [63]. Miech et al. [20] has furthermore concluded that different mental disorders are differently related to social position, which points to the importance of research with measures of specific mental disorders and not overall measures of poor mental health.

There are likely several mechanisms behind the social differences, which are not explored in the current study. There are many different determinants for depression, such as negative social circumstances including low social support and poverty [21, 22], and serious conflict during upbringing [64]. Differential exposure to these determinants can cause inequalities. One additional explanation is that of susceptibility, meaning that some social groups are more vulnerable. Long lasting stress is one factor that can contribute to depression [65] but the same amount of stress can affect people differently. Higher SES groups may have better opportunities for social support and know where to turn for help in handling stress. Furthermore, negative psychosocial factors can cluster in persons with low social position and etiological mechanisms can differ depending on at what age depression is present. For example, juvenile onset depression is more affected by adverse childhood factors than adult onset depression [66].

### Strengths and limitations

The possibility to use register-based material, encompassing a large population-based sample of adolescents with both in- and outpatient data, is a great strength of the current study. Including both in- and outpatient data has been found to be important to calculate reliable rates of mental diseases [67]. Population characteristics and the distribution of care utilities are similar to other big cities of Sweden and thus it is possible to generalize to those, as well as to other populations within a similar context. The cohort design allowed an etiological focus and the clinical assessment of diagnoses within CAMHS offered a naturalistic design. Furthermore the diagnostic procedures at CAMHS are based on professional assessment and not on structured instruments to measure depressive symptoms. The use of register-based material decreased problems with self-reported data such as recall bias. Swedish official registers have overall a high standard and are useful for research purposes [68]. Concerning depression, a validation study in Sweden among adults showed 88% agreement when depression diagnosis from out-patient care was compared to a clinical diagnosis from court-ordered inpatient evaluations [69].

One important limitation of this study is possible misclassification of the outcome, where some adolescents who fulfil the criteria of depression are classified as not having a depression. This can be due to that adolescents with depression often do not seek care [70], reluctance by care givers to diagnose children [71], as well as the possibility of misdiagnosis. The incidence presented may therefore underestimate the true levels of depression in the population. Potential social differences in care-seeking introduce further bias [9] though the direction of the bias is not clear. Finnish studies [14, 72] suggest that children of parents with higher socioeconomic position may have better access to care. Differences in care seeking can also apply to gender and ethnic background, where boys in later adolescence and those with parents from ethnic minorities are less likely to seek care [73]. This would result in an underestimation of the risk for those groups in the current study. On the other hand some care in the private sector was not included in the current analyses, and if those with higher social position to a higher extent seek private care this would lead to an overestimation of the results.

Another possible, but less severe, limitation is misclassification of exposure due to the procedures for registering housing. Children can only be registered at one address, thus only one social context is considered for those with alternate living arrangements. Furthermore, in the current study the second parent was excluded if he or she was not a biological/adoptive parent, since information for those was differential due to housing conditions, which in turn can be related to social position. This misclassification is not likely to be large but can classify adolescents to a lower social position than they should be, and thus may lead to an underestimation of the associations.

A history of own major depression has been found to confound the relationship between SES and major depression among adults [41] but parental depression only marginally affected the results in the current study, which is in line with findings from Modin et al. [74]. Other measures of poor parental mental health could have been used as also other mental disorders can have an impact on SES and offspring depression. One additional measure of parents’ mental disease, defined as any mental disorder severe enough for in-patient care, was tested in the current study. Including this as a confounder changed the estimates marginally compared to adjusting for depression. Childhood adversities and psychosocial factors were considered mediators in the current study as they are within the causal pathway between social position and depression. Patterson et al. [75] imply for example that adversities in youth increase the risk of depression more than family history of parental mental disease do, and that the adversity is more related to SES.

### Implications

The social differences detected in the current study were modest, and still more work needs to be done to reduce social differences. This is especially important considering that a great number of people are affected by depression, and to a higher extent in groups with lower social position. Disentangling the role of social position in care-seeking can further guide the direction of future research, access to care and mental health promotion.

## Conclusion

Social differences in diagnosed depression were found for adolescents in Sweden. The differences were modest and gender differences negligible.
